# Using molecular approaches to assess rabies virus diversity in Haiti and the Dominican Republic

**DOI:** 10.3389/fmicb.2025.1688184

**Published:** 2026-01-20

**Authors:** Rene E. Condori, Augustin Pierre Dilius, Rolain Cadet, Griselda Lopez-Nuñez, Elinna Diaz-Mateo, Anna K. Gomez-Belliard, Yasmeen B. Ross, Cassandra Boutelle, Andres Velasco-Villa, Crystal M. Gigante, Yu Li, Ryan M. Wallace

**Affiliations:** 1 Poxvirus and Rabies Branch, Division of High Consequence Pathogens and Pathology, National Center for Emerging and Zoonotic Infectious Disease, Centers for Disease Control and Prevention, Atlanta, GA, United States; 2 Golbelt Professional Services, Chesapeake, VA, United States; 3 Ministry of Agriculture, Port-au-Prince, Haiti; 4 Laboratorio Nacional de Salud Pública Dr. Defilló, Ministerio de Salud Pública y Asistencia Social, Santo Domingo, Dominican Republic; 5 Centro de Prevención y Control de Enfermedades Transmitidas por Vectores y Zoonosis (CECOVEZ), Ministerio de Salud Pública y Asistencia Social, Santo Domingo, Dominican Republic; 6 Dirección Provincial de Salud Santiago, Ministerio de Salud Pública y Asistencia Social, Santo Domingo, Dominican Republic

**Keywords:** rabies, phylogenetic, Caribbean, Haiti, Dominican Republic

## Abstract

Hispaniola, comprised of Haiti and the Dominican Republic, is a rabies-endemic island. Since 2013, Haiti and the Dominican Republic have operated advanced surveillance systems that have increased the number of humans and animals tested. In this study, rabies-positive samples collected between 2014 and 2023 were sequenced. Phylogenetic analysis revealed that the rabies virus (RABV) from Hispaniola forms a novel ‘Caribbean subclade’ with two distinct lineages: a major lineage associated with dogs and found across Haiti and parts of the Dominican Republic and a second lineage associated with dogs and mongoose found only in the Dominican Republic. Phylogenetic data support dogs as the main rabies reservoir in Haiti and show evidence of cross-border transmission. In the eastern part of the Dominican Republic, the evidence supports mongooses as the primary reservoir of rabies. Subclades within Haiti suggest that geospatial segregation of dogs and rabies may offer opportunities for zoned rabies elimination programs.

## 1 Introduction

Despite the availability of effective vaccines for animals and people, rabies continues to be neglected in many low- and middle-income countries in Africa, Asia, and Latin America (Vigilato et al., 2013; Taylor and Nel, 2015). In rabies-endemic countries, limited vaccine availability, poor access to healthcare among marginalized populations, and insufficient laboratory capacity to confirm suspect cases are major barriers to elimination and contribute to the persistence of the disease (Fahrion et al., 2017; Rajeev et al., 2020; Swedberg et al., 2024). After government-sponsored initiatives to control rabies were widely adopted in the 1980s across Latin America and the Caribbean, prevention and surveillance systems were implemented to reduce the incidence of human rabies. Although these efforts have been largely successful, dog-mediated rabies still persists in several countries—including Bolivia, Brazil, Peru, Venezuela, Argentina, Colombia, Guatemala, Cuba, Haiti, and the Dominican Republic (Meske et al., 2021; Seetahal et al., 2018). During 2022 and 2023, the Regional Information System for the Epidemiological Surveillance of Rabies (SIRVERA), which tracks rabies cases in Latin American countries, recorded six human cases of rabies transmitted by dogs in Venezuela, Peru, Bolivia, and Haiti (Pana American Health Organization, 2024). However, this likely underestimates the true burden and underscores that dog-mediated rabies deaths continue to occur in the Western Hemisphere.

Haiti, a country located in the Caribbean, has a fragile health infrastructure, with health services severely constrained (Lowrance et al., 2017). Therefore, rabies is enzootic in Haiti, with estimates suggesting approximately 50 to 130 human rabies cases per year, depending on the availability of human and dog rabies vaccines (Wallace et al., 2017; Kunkel et al., 2021; Wallace et al., 2015; Freire de Carvalho et al., 2018). In contrast, in the neighboring Dominican Republic, implementation of large-scale dog vaccination programs has contributed to significant reductions in human and dog rabies cases (Velasco-Villa et al., 2017). Despite the fact that dog-mediated rabies is almost under control in the Dominican Republic, there are still sporadic rabies outbreaks and human deaths (Mandra et al., 2019). Another relevant rabies reservoir species in the Caribbean is the Indian mongoose (*Herpestes auropunctatus*). Rabies in mongoose in the Western Hemisphere was first documented in Puerto Rico in the 1950s, after the introduction of rabies in dogs and a subsequent host shift to the mongoose (Tierkel et al., 1952). Since then, there have been recurrent outbreaks of different magnitudes across several Caribbean islands (Nadin-Davis et al., 2006; Zieger et al., 2014). In the Dominican Republic, this species gained importance in the 1980s, when 139 mongoose were confirmed to be rabies-positive in the laboratory, suggesting that they may be acting as a reservoir in the country (Everard and Everard, 1992). The presence of mongoose-maintained rabies viruses (RABVs) in Haiti has never been documented; however, the small Indian mongoose is endemic across the island of Hispaniola, raising the question of whether the mongoose may be a rabies reservoir on Hispaniola and, if so, whether rabies affects the mongoose population across the island. A 1992 genetic study of the rabies virus, which included human, dog, and two mongoose samples from the Dominican Republic, found that the rabies virus (RABV) detected in the Dominican Republic was genetically close to the RABV detected in Puerto Rico and Peru. However, limited genetic studies have been conducted to better understand the origins, reservoir implications, and distribution of rabies viruses across Hispaniola (Smith et al., 1992).

Since there is no physical barrier that can prevent rabies movement between countries, detailed molecular studies can help identify isolated pockets of rabies, which can be used to inform rabies control and elimination strategies, particularly in countries such as Haiti and the Dominican Republic, which have invested in rabies vaccination programs for several decades. Therefore, the aim of this study was to determine the genetic diversity of RABV variants circulating in Haiti and the Dominican Republic, as well as to identify their geographic distribution and the potential rabies reservoir host(s) associated with them.

## 2 Materials and methods

### 2.1 Origin of isolates

Surveillance activities in Haiti were approved under IACUC 2929DOTMULX-A5, and samples from the Dominican Republic were collected as part of routine rabies surveillance.

A total of 92 brain tissue samples were collected in Haiti (dog = 89, cat = 1, goat = 1, and human = 1) from 8 of 10 departments, while 40 samples were collected in the Dominican Republic (dog = 23, human = 6, cat = 6, mongoose = 3, bovine = 1, and horse = 1) from 16 of 31 provinces (Table 1). In addition, we included a RABV sequence obtained from a human rabies case in a traveler who was exposed to the rabies virus in Haiti but was diagnosed in the USA (Centers for Disease Control and Prevention C, 2012). All samples included in this study were submitted to the CDC’s National Rabies Reference Laboratory for genetic characterization. Initially, the samples were tested using either the direct fluorescent antibody (DFA) test (World Organisation for Animal Health O, 2018) or the real-time RT-PCR LN34 assay (Wadhwa et al., 2017). Detailed information for each sample can be found in Supplementary Table 1.

**Table 1:** Geographic origin and total number of samples included in this study from Haiti and the Dominican Republic.

Haiti		Dominican Republic	
Department	No. samples	Province	No. samples
Artibonite	5	Dajabon	4
Centre	18	Distrito Nacional	2
Grand’ Anse	1	Duarte	2
Nord	6	El Seibo	1
Nord Est	1	Elias Piña	3
Nord Ouest	1	Independencia	1
Ouest	45	La Altagracia	2
Sud	4	La Vega	1
Unknown	11	Monte Plata	1
		Pedernales	4
		Puerto Plata	5
		San Cristobal	2
		San Pedro de Macoris	1
		Santiago	2
		Santo Domingo	5
		Valverde	1
		Unknown	3
Total	92		40

*The geographic resolution for each sample was at the department or province level.

### 2.2 RNA extraction and phylogenetic analysis

Total RNA was extracted from the rabies-infected brain tissue using TRIzol® (Life Technologies) with MagNA Lyser Green Beads (Roche) and homogenized in a Mini BeadBeater (BioSpec Products). After centrifuging the samples for 4 min at 14,000 x g, 300 μL of the collected supernatant was mixed with an equal volume of molecular biology-grade ethanol (200 proof). Total RNA was extracted using the Direct-zolTM RNA MiniPrep Kit (Zymo Research Corporation), and the protocol can be found at https://www.protocols.io/private/5c970341ebdf05cba17e58ebc16dff08. Details of molecular testing and phylogenetic analysis are provided in the Supplementary material.

## 3 Results

### 3.1 RT-PCR and phylogenetic analysis

**Figure 1:**
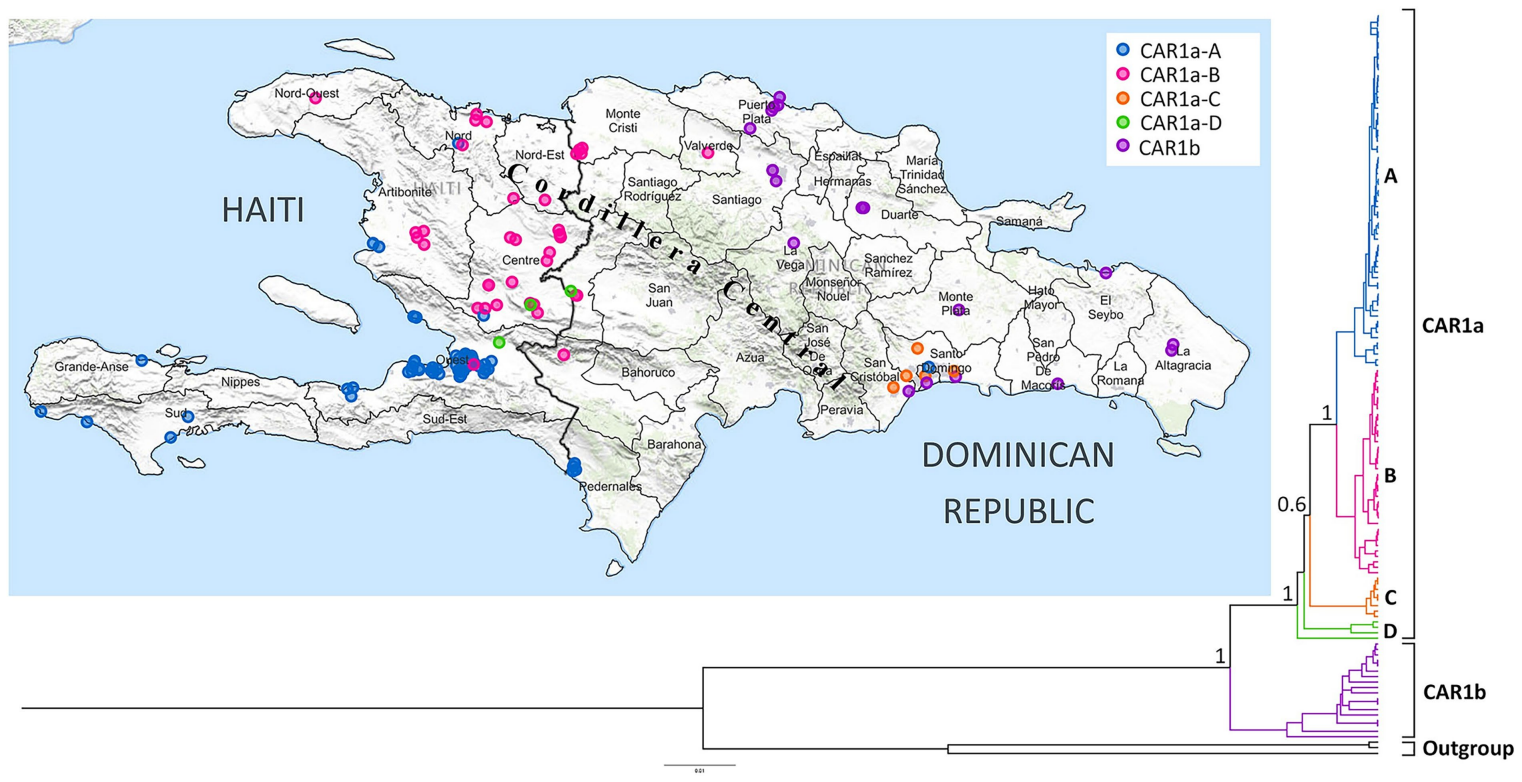
Map showing the geographic distribution of the RABV samples collected across Haiti and the Dominican Republic, with the accuracy of the location at the city level. The colors correspond to the respective lineage/sub-lineage in the phylogenetic tree. The phylogenetic tree was constructed using the complete and partial N gene datasets in BEAST 2.5. A total of two major lineages—CAR1a and CAR1b—were determined, and the sub-lineages A to D of CAR1a are displayed in different colors. The numbers at the main branch nodes represent the posterior probabilities that support rabies lineages/sub-lineages. The number in the scale bar represents the nucleotide substitution per site.

A total of 132 complete nucleoprotein (N) gene sequences and one partial N gene sequences were generated. The phylogenetic inferences conducted using strict and relaxed clocks in BEAST 2.5 produced similar phylogenetic trees and revealed two main lineages with distinct geographic distributions, hereafter referred to as Haiti–Dominican Republic (CAR1a) and Dominican Republic (CAR1b) (Figure 1).

The CAR1a lineage contained 115 sequences (Haiti = 93, Dominican Republic = 22). Most of the sequences corresponding to this lineage were obtained from the samples collected from the Ouest and Centre departments of Haiti. The CAR1a lineage diverged into four sub-lineages (Figure 1). Sample details and GenBank accession numbers can be found in Supplementary Table 1.

Most sequences from CAR1a-A (65 samples: Haiti = 60, Dominican Republic = 5) were from the Haitian Ouest department (n = 44), as well as several departments in the southern half of the country and a bordering province of Pedernales in the Dominican Republic (four samples). A single sequence of this sub-lineage was found in the city of Guaricano in the Santo Domingo province in the Dominican Republic, approximately 315 kilometers from the nearest case in this sub-lineage. This finding likely suggests a human-mediated translocation. Although dogs were the predominant species within this sub-lineage, a goat and four human cases were also identified.

Sub-lineage CAR1a-B contained 38 isolates (Haiti = 30, Dominican Republic = 8). The Centre department in Haiti contributed the majority of sequences for this sub-lineage (*n* = 16). Other members of CAR1a-B were found in the neighboring Haitian departments of Artibonite (*n* = 4), Nord (*n* = 5), Nord Ouest (*n* = 1), Nord Est (*n* = 1), and Ouest (*n* = 1), and the locations for two sequences were unavailable. Within the Dominican Republic, CAR1a-B was found in Dajabon (*n* = 4), Elias Piña (*n* = 2), Independencia (*n* = 1), and as far as the province of Valverde (*n* = 1). Animals identified with CAR1a-B included 36 dogs and two cats.

Sub-lineage CAR1a-C contained eight sequences from the Dominican Republic collected between 2009 and 2012. The locations for three sequences were not available, while the remaining five sequences were distributed across the provinces of Santo Domingo, San Cristobal, and Distrito Nacional. Species identified with CAR1a-C included five dogs, two cats, and one human.

Sub-lineage CAR1a-D contained four sequences (Haiti = 3, Dominican Republic = 1) collected from dogs. This sub-lineage was genetically diverse and may comprise two sub-lineages, which could become more evident when more data becomes available.

The CAR1b lineage contained 18 isolates from the Dominican Republic and was widely distributed, from the northeast province of Puerto Plata to the southern provinces of La Altagracia and Santo Domingo. It included 12 domestic animals, three humans, and three mongooses. One human and two dog cases were epidemiologically linked to a mongoose bite.

**Figure 2:**
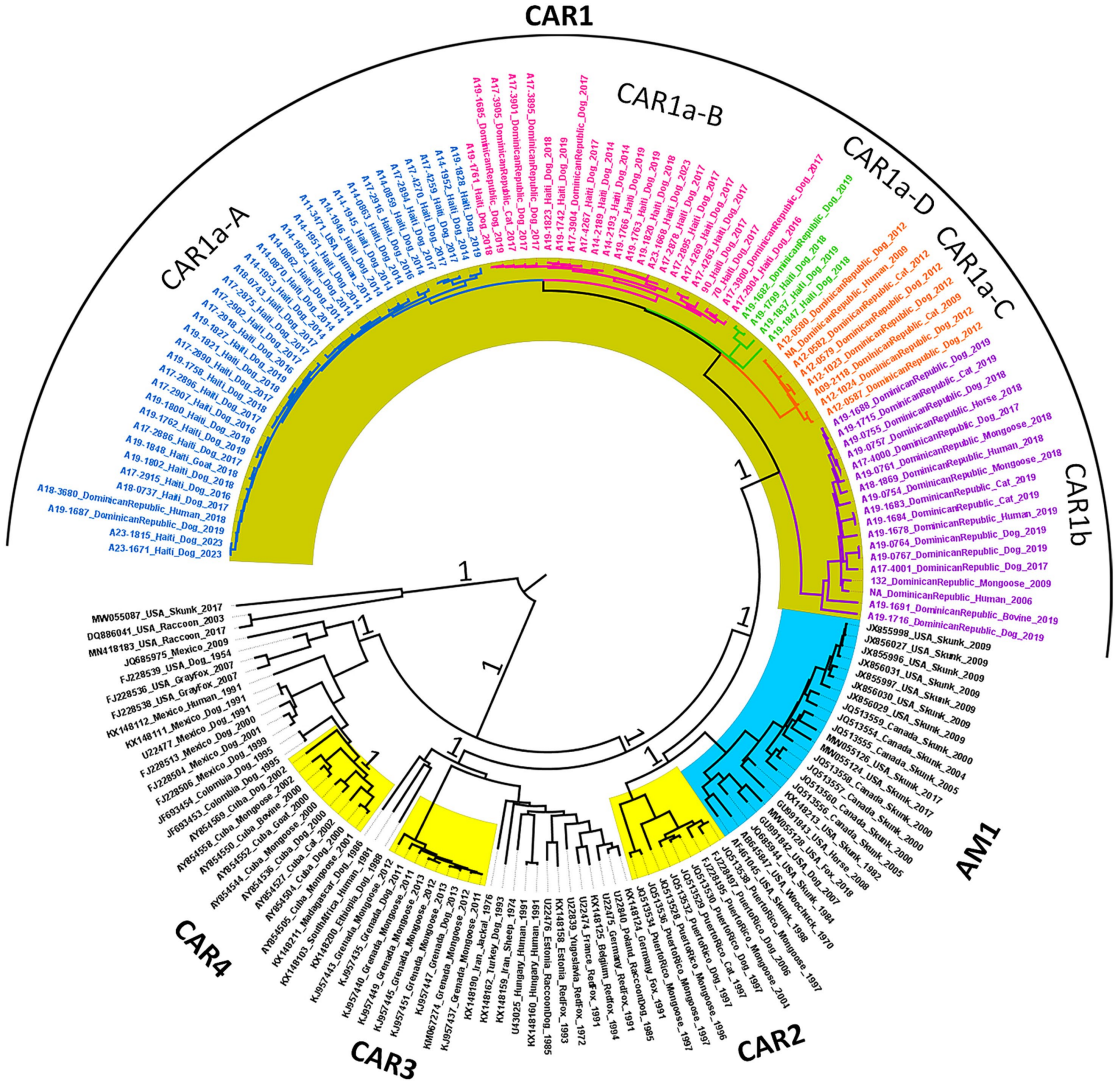
Phylogenetic tree of the complete N gene sequences of the RABV samples from Hispaniola, along with representative isolates from the Caribbean and other major rabies strains of the Cosmopolitan clade. The Caribbean 1 (CAR1) subclade is highlighted in gold. The colored branches indicate the CAR1a and CAR1b lineages. The CAR1a lineage diverged into four sub-lineages, which are identified by colors in the taxon labels. The branches highlighted in sky blue represent the North Central Skunk (NCSK) rabies variant or AM1, and the branches highlighted in yellow represent the RABVs detected in other Caribbean islands. The numbers at the nodes indicate the posterior support for each clade.

A phylogenetic reconstruction using a representative subset of 90 complete N gene sequences from Hispaniola and 81 reference sequences representing major clades associated with dog-mediated rabies globally (Supplementary Table 2) demonstrated that all RABV sequences generated for this study belonged to the Cosmopolitan clade (Troupin et al., 2016). The Hispaniola sequences formed an independent subclade that shares a common ancestor with RABVs found in mongoose and dogs in Puerto Rico, as well as with the AM1 subclade, which includes RABV sequences primarily associated with striped skunks in the north-central region of the continental USA and southern Canada. RABVs detected in other Caribbean islands, such as Cuba and Grenada, branched distantly. Aiming to differentiate RABVs from Hispaniola from other dog-mediated RABV clades and lineages in the Western Hemisphere, we designated them as the Caribbean 1 (CAR1) subclade, which further splits into two lineages: the CAR1a lineage with four sub-linages, and the CAR1b lineage. The RABVs from Puerto Rico were identified as CAR2, those from Grenada as CAR3, and those from Cuba as CAR4 (Figure 2).

**Figure 3:**
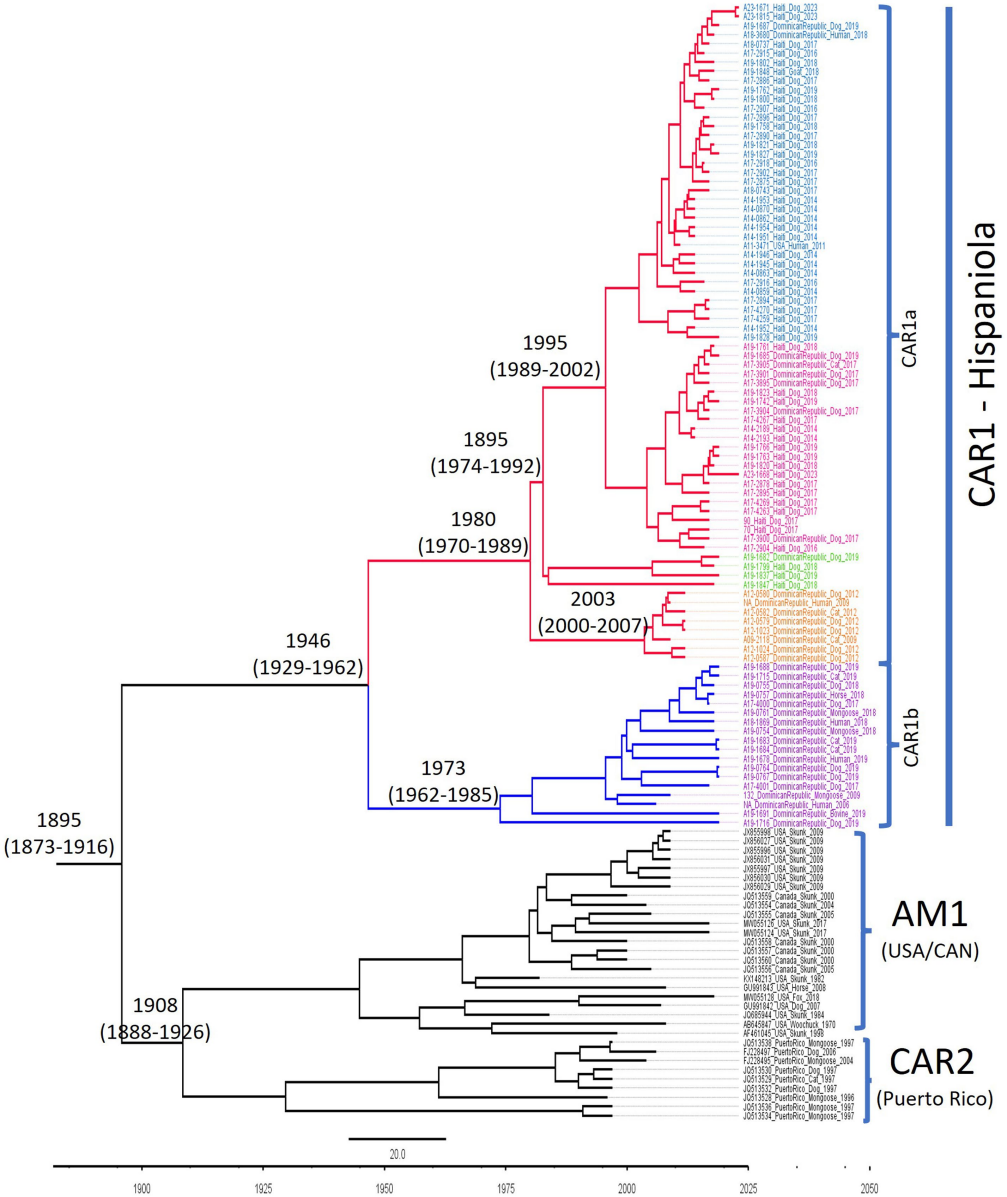
Phylogenetic tree generated using the RABV sequences from Hispaniola, along with the representative sequences of the NCSK rabies variant from North America and RABVs from Puerto Rico. The years at the node indicate the estimated time of the most recent common ancestor (TMRCA); the scale bar represents time in years (25 years).

The Hispaniola subclade CAR1 diverged from its most recent common ancestor with CAR2 and AM1 around 1895 (95% HPD 1873–1916) (Figure 3). The RABVs included in this study diverged into lineages CAR1a and CAR1b around 1946 (95% HPD 1929–1962). The lineage CAR1a, which became established in the western part of the island, subsequently split into four sub-lineages around 1980 (95%: HPD 1970–1989). Sub lineage CAR1a-C, which was detected only in the Dominican Republic, emerged around 2003 (95% HPD: 2000–2007), and the two major sub-lineages A and B of CAR1a diverged around 1995 (95% HPD: 1989–2002) The CAR1b lineage emerged around 1973 (95% HPD: 1962–1985). The estimated substitution rate was 2.86 × 10^−4^ substitutions/site/year (95% HPD of 2.14 × 10^−4^ to 3.63 × 10^−4^).

The partial RABV genome sequences(200 bp) from a human and a dog in Boca Chica municipality, Dominican Republic, clustered within the CAR1a-C sub-lineage, while the RABV sequences from two mongooses in La Vega and Bayaguana municipalities were placed in the CAR1b lineage (Supplementary Figure 1).

We observed that most isolates from mongooses in Grenada and Puerto Rico, as well as those clustered in CAR1b, carried a unique mutation at position 254. The RABVs from Grenada and Puerto Rico, which diverged from the former dog-maintained variant, presented a lysine at position 254, while the sequences from the Dominican Republic that clustered in the CAR1b lineage presented a glycine in 17 of 18 sequences. All other reference sequences associated with dogs or other terrestrial carnivores presented an arginine at position 254.

## 4 Discussion

The first known introductions of the Cosmopolitan RABV clade from the Old World into the Western Hemisphere took place during the period of European colonization in the 15th and 16th centuries, with no conspicuous flourishment or establishment (Holtz et al., 2023). It was not until the late 18th and 19th centuries, after better-connected and more densely populated colonies were established, that dog-mediated rabies effectively spread throughout the Americas and Caribbean islands (Velasco-Villa et al., 2017). In certain environments, the Cosmopolitan clade established itself and transitioned into new reservoir species, such as mongooses, skunks, and foxes (Acharya et al., 2022). Although these host shifts from dogs to wildlife are estimated to have taken place at least two centuries ago, rabies viruses from these host shifts continue to exhibit a significant antigenic and molecular resemblance to their ancestral Cosmopolitan RABV strains that once circulated in dogs (Nadin-Davis et al., 2006; Zieger et al., 2014; Nadin-Davis et al., 2008).

In this study, we analyzed the complete and partial N gene sequences of the samples collected in Haiti and the Dominican Republic through routine surveillance activities. Our study is the first of its kind to include samples collected from multiple geographic locations across Hispaniola and the first such analysis to be conducted in over 30 years. The analysis of the N gene provides an initial view of rabies diversity, and previous studies have shown that results are often consistent with those from complete rabies genome analysis, which is more complex and time-consuming. The phylogenetic analysis presented here identified two RABV lineages that are enzootic in Hispaniola: one primarily distributed throughout Haiti, which we designated as the CAR1a lineage and which further split into four sub-lineages, one of which was entirely unique to the Dominican Republic. Most samples collected in Haiti came from the departments of Ouest and Centre, which have received consistent financial and technical support from the CDC for surveillance and sample collection, likely leading to an over-representation of samples from these locations (Wallace et al., 2017).

In 2013, with the aim of reducing the number of human rabies cases, the Haiti Animal Rabies Surveillance Program (HARSP) was implemented and routine surveillance was improved, which contributed to the detection of an increased number of rabid animals in the Ouest, Centre, Artibonite, Nord, and Sud departments (Millien et al., 2023; Schrodt et al., 2023). The magnitude of the rabies surveillance effort of the HARSP can be observed in the RABV diversity identified in this study. In the future, improving rabies surveillance in departments with limited capacity may reveal additional RABV lineages and provide further insights into rabies diversity across the island of Hispaniola.

### 4.1 Haiti and the Dominican Republic rabies virus lineages

The ancestor of the Haiti and Dominican Republic lineage (CAR1a) emerged around 1980 (Figure 3) and subsequently split into four sub-lineages. CAR1a-A and CAR1a-B were the most prevalent and comprised the majority of the Haitian samples. The CAR1a-A hotspot consisted primarily of rabid dogs located in urban centers near the western coast of the Ouest department (Port au Prince) and was the only sub-lineage detected in southern Haiti, while CAR1a-B was predominantly distributed across smaller cities within Centre. CAR1a-A and CAR1a-B split from a common ancestor around 1995, a period marked by intense political instability in Haiti that led to reduced human movements between the northern and southern regions of the country. Furthermore, there are several large mountain ranges that span longitudinally across Haiti near the northern extent of CAR1a-A. The combined effects of political sequestration of human populations (and consequently their dogs) and geological features that hinder natural dog movement may have resulted in the formation of this unique sub-lineage.

From a rabies management perspective, identifying sequestered pockets of rabies circulation may provide valuable insights for implementing interventions such as mass dog vaccination campaigns. Targeted efforts to eliminate a single sub-lineage may be successful even if control of other lineages is not simultaneously achieved; however, this may leave a vacuum for reintroduction at a later time.

Between 2009 and 2012, five of eight RABV sequences belonging to CAR1a-C were identified around Santo Domingo and San Cristobal in the Dominican Republic. After 2012, no new sequences were identified anywhere across the island of Hispaniola, suggesting that this sub-lineage may have been eliminated through the Dominican Republic’s rabies control program, which was first implemented in the 1960s (Everard and Everard, 1992). Elimination of dog-mediated rabies variants/lineages is usually achieved through large-scale dog vaccination programs and efforts to reduce the density of free-roaming dogs (Fisher et al., 2018; Puebla-Rodríguez et al., 2023).

A total of four genetically diverse RABV sequences formed CAR1a-D, a group that overlapped spatially and temporally with CAR1a-A and CAR1a-B. Within CAR1a-D, one sequence (A19-1847 Haiti_Dog_2018) from the Ouest department was genetically divergent from other members of the CAR1A lineage. Due to limited sampling, we cannot rule out the possibility that these four sequences may ultimately form two additional sub-lineages, with a distinct geographic distribution, but this may also represent viral sequestration and divergence within the highly fragmented and mountainous region of central Hispaniola.

### 4.2 The Dominican Republic mongoose lineage CAR1b

Our analysis estimated that the CAR1b lineage in the Dominican Republic split from CAR1a around 1946 (Figure 3). Around the time of this genetic branching, the country experienced increasing political repression and extensive expansion of sugarcane plantations, a prime habitat for mongooses, which were still used for pest control at that time. While it is impossible to determine definitively, the environmental and political changes at the time of the emergence of CAR1b may have led to a viral host shift to a then-growing and robust mongoose population, coupled with increased interactions with dogs.

The importance of mongooses as a potential rabies reservoir on the island of Hispaniola was first recognized in the 1980s (Everard and Everard, 1992). The CAR1b lineage shows strong epidemiological links to mongooses, suggesting that this species is likely the viral reservoir. During the timeframe of this study, CAR1b was identified from the province of Puerto Plata in the north to La Altagracia in the southeast, including Santo Domingo, suggesting that this lineage may be geographically constrained to the eastern side of the “Cordillera Central” mountain range, which extends from San Cristobal province in the southern Dominican Republic to northeast Haiti.

Elevations of >300 meters are considered a barrier to mongoose dispersal; therefore, it is possible that the geographical features present in the Dominican Republic may be helping to deter or delay the westward expansion of the CAR1b lineage, which includes rabies cases in mongooses (Berentsen et al., 2018; Sauve et al., 2021). A study that analyzed raccoon rabies suggested that natural barriers such as rivers and forests may be involved in reducing the spread of raccoon rabies in the USA; however, even these barriers often fail due to ecosystem changes or human-mediated animal movements (Smith et al., 2002).

Rabies in mongooses has not been investigated in depth throughout Haiti; therefore, we cannot rule out the possibility that mongooses may serve as a reservoir in Haiti. However, domestic dogs remain the most significant reservoir responsible for human rabies cases. Once rabies is eliminated in domestic dogs, rabies circulation in wildlife reservoirs may become more easily detectable. Enhanced rabies surveillance in mongooses is needed to determine the magnitude and distribution of rabies outbreaks in this species and may help reveal the emergence of new RABV variants or lineages, which can contribute to the early detection of geographic expansion events. The discovery of a distinct rabies virus variant in wildlife species is critical for informing rabies control strategies, and continued viral characterization is important to determine whether rabies cases result from viruses maintained in dogs or wildlife. Certainly, in this study, almost all cases that involved the CAR1a lineage were detected only in dogs, while the CAR1b lineage included RABV isolates from mongooses, humans, and other domestic animals, several of which had known mongoose exposures.

### 4.3 Rabies virus translocation events

Human migration patterns, along with commerce and transportation networks, offer anthropogenic pathways of moving viruses and viral reservoirs across large distances (Burgos-Cáceres, 2011; Sararat et al., 2022; Talbi et al., 2010; Colombi et al., 2020; Dellicour et al., 2017). In this study, several geographic and transboundary translocation events were identified within the CAR1a lineage. In the southern part of Hispaniola, located around 137 km from the epicenter of sub-lineage CAR1a-A in Haiti, four human rabies deaths associated with this sub-lineage were detected in the city of Pedernales, Dominican Republic (Mandra et al., 2019). In addition, another member of CAR1a-A was detected in Santo Domingo, approximately 200 km from the city of Pedernales. A member of sub-lineage CAR1a-B was identified in the town of Hato Nuevo-Mao in the province of Valverde, which is located approximately 67 km from the Haitian border, where most cases of this sub-lineage were observed.

The detection of members of sub-lineage CAR1a-A and CAR1a-B in areas distant from their likely enzootic regions may reflect human-mediated translocation of rabies-infected animals or gaps in surveillance in the western Dominican Republic. Therefore, we cannot rule out a broader geographic distribution of these sub-lineages beyond what was identified in this study. However, the sporadic detection of this lineage in the Dominican Republic, far from its likely enzootic range in Haiti, along with relatively robust veterinary health systems in the Dominican Republic, indicates that human-mediated movement of infected dogs occurs in Hispaniola and jeopardizes decades of rabies control efforts on the island. Understanding rabies dynamics in dogs across the border between Haiti and the Dominican Republic is essential to monitor dog rabies reintroduction, and the findings of this study emphasize the importance of bi-national rabies control efforts.

### 4.4 TMRCA of the Hispaniola subclade and its relationship with AM1 and other Caribbean subclades

The extended phylogenetic analysis, which included representative RABV strains, confirmed that RABVs circulating on the island of Hispaniola are part of the Cosmopolitan clade with strong posterior support. The estimated substitution rate was 2.86 × 10^−4^ substitutions/site/year (95% HPD of 2.14 ×10^−4^ to 3.63×10^−4^), consistent with previously reported values (Holmes et al., 2002; Biek et al., 2015; Layan et al., 2021). The RABV from Hispaniola, named CAR1 in this study, shared a common ancestor with the rabies virus variant found in skunks in the North Central USA, which was named AM1 (Troupin et al., 2016), and RABV circulating in Puerto Rico (CAR2) (Figure 2). Other rabies strains on the Caribbean islands are genetically different. For example, the Cuban strain (CAR4) is closely related to the Mexican Dog variant (Nadin-Davis et al., 2006), while the rabies found in Grenada (CAR3) is closely related to strains from certain European regions (Zieger et al., 2014).

In our analysis, the TMRCA of the AM1 and CAR2 subclades was estimated to have occurred around 1908 (Figure 3). Considering that no sequences from Hispaniola were available prior to our study, the precise timing of rabies introduction on the island of Hispaniola is unknown, but the first documented cases were reported around 1788 (Millien et al., 2023).

Our analysis focused on the nucleoprotein gene, which allowed us to cover a broader geographic and temporal range of rabies virus sequences. The nucleoprotein gene is the most extensively studied gene in the rabies virus and has been used for phylogenetic analysis for decades, meaning that most historical isolates were sequenced for either the partial or complete nucleoprotein gene. While whole genome sequencing can provide higher resolution, it is not practical for rabies surveillance in resource-limited settings, as its cost is much higher and there is no validated approach for viral enrichment across the diversity of rabies viruses. While most sequences available for AM1 are complete or partial nucleoprotein sequences, some studies have produced rabies virus phosphoprotein gene sequences from Puerto Rico and Cuba. Our estimate of the TMRCA of AM1 and CAR2 (1908, Figure 3) agrees with Troupin et al.’s analysis, which was based on complete and partial rabies genomes (Troupin et al., 2016). However, a recent analysis determined that the parental TMRCA of AM1 was around 1810 (Holtz et al., 2023). In the future, when complete genome sequences from Hispaniola become available, the TMRCA of AM1 may be adjusted. The findings of this study suggest that rabies in Puerto Rico and Hispaniola each descended from a common colonial-era ancestor, likely introduced to each island separately in the late 18th or early 19th centuries, and subsequently spread among dogs and local wildlife (Velasco-Villa et al., 2008).

## 5 Conclusion

Rabies elimination efforts in the Western Hemisphere have been largely successful by leveraging supportive government policies to implement widespread dog vaccination programs. However, several niduses of dog-mediated rabies remain and threaten to undermine these successes. Haiti and the Dominican Republic represent two such countries where dog-mediated rabies cases persist despite decades of government control efforts.

The molecular epidemiologic findings in this study provide new insights into the current dynamics of rabies in Hispaniola and potential control strategies. Haiti has multiple dog-maintained RABV lineages that show geographic separations, potentially allowing for staged, lineage-targeted rabies control efforts. This approach may help address challenges inherent in low-resource countries.

The Dominican Republic is likely approaching the elimination of dog-maintained rabies, but the virus appears to be established in local mongoose populations. This study clearly shows that political boundaries do not prevent RABV movement and that rabies control in Haiti and the Dominican Republic is inextricably linked; therefore, bi-national approaches offer a better pathway to island-wide rabies elimination.

## Data Availability

Rabies virus nucleoprotein sequences for this study can be found in the GenBank repository with accessioning number PX738125 to PX738257.
