# Supplementary material for: Using molecular approaches to assess rabies virus diversity in Haiti and the Dominican Republic
Source: Front Microbiol. 2026 Jan 20;16:1688184. doi: 10.3389/fmicb.2025.1688184 (PMC12864505; doi:10.3389/fmicb.2025.1688184)
Supplement: Supplementary file 1 [file Data_Sheet_1.docx]

Supplementary Material

1. **Material and Methods**
   1. **LN34 testing, RT-PCR, and nucleotide sequencing.**

The LN34 pan-lyssavirus real-time RT-PCR assay was employed to confirm RABV nucleic acid (1, 2). To generate the complete N gene for samples collected between 2009 to 2017, complementary strand DNA (cDNA) was produced by using a reverse transcriptase AMV (10109118001 Roche) with a specific forward primer LYS001(3). dsDNA was amplified by the standard polymerase chain reaction (PCR) using a combination of primers (4). PCR products were purified using ExoSAP-IT^TM^ PCR Product Cleanup Reagent (78205 Applied Biosystems). Sanger sequencing was achieved by using BigDye Terminator v1.1 Cycle sequencing kit (4337452 Applied Biosystems) and amplicons were sequenced in ABI 3730 DNA analyzer (Applied Biosystems).

For samples collected between 2018 to 2023, the complete N gene was amplified using a SuperScript™ IV One-Step RT-PCR System (12594100 Invitrogen) with primers NgeneFor and NgeneRev (5). The 20 µL reaction mix included 10 µL of 2X Platinum SuperFi RT-PCR Master Mix, 0.5 µL of forward and reverse primers (20 uM),0.2 µL of SuperScript IV RT Mix, 5 µl of RNA, and 4 µL of DNase/RNase-Free Water. The RT-PCR conditions were 50 °C for 10 min followed by 98 °C for 2 min and 35 cycles of 98 °C for 30 s, 56 °C for 30 s, 68 °C for 2 min 30 s with a final 5 min extension at 72 °C. A total of 5 µL of cDNA was mixed with 2 µl of ExoSAP-IT™ *Express* PCR Product Cleanup Reagent (75001 Applied biosystems).

A Takara LA Taq Polymerase with GC buffers kit (RR02AG Takara) was used in the barcoding PCR. 5 µL of purified cDNA was added to the PCR reaction mix containing 10 µL of 2x GC buffer I, 2 µL of dNTPs, 0.2 µL of Takara LA Taq, 2.3 µL of nuclease-free water and 1 µL of unique barcode designed by Oxford Nanopore Technologies (EXP-PBC096) was added to each sample. Barcoding PCR conditions were 94 °C for 1 min followed by 14 cycles of 94 °C for 30 s, 62 °C for 30 s, 72 °C for 2 min, and a 5 min final extension at 72 °C. DNA concentration of each barcoded samples was quantified using a Qubit dsDNA HS (High Sensitibity) assay (Q32851 Invitrogen). 200 ng of each sample was pooled and clean-up using 0.65X AmpureXP (A63881 Beckman). 500 ng of pooled DNA was used for the library preparation following the instructions for the genomic DNA ligation kit (SQK-LSK109 Oxford Nanopore Technologies), sequencing was run for 24 hours on a MinION sequencer using a flongle adapter (Oxford Nanopore Technologies) and FLG001 flowcells with >70 pores.

- 1. **Sequence analysis and phylogenetic reconstruction.**

Complete N gene (1350bp) sequences obtained by sanger sequencing were assembled and edited using Bioedit 7.0.5.3 (6). Samples sequenced using the Oxford nanopore technology were analyzed by basecalling and demultiplexing on guppy v6.1.2 requiring barcodes at both ends; sequence reads were mapped to reference sequences in minimap2 pomoxis v0.1.0 (<https://nanoporetech.github.io/pomoxis/>); the consensus sequences were extracted using ivar v0.1 (<https://andersen-lab.github.io/ivar>) -t 0.6 -m 55 and then polished using medaka v.1.0.1 (<https://github.com/nanoporetech/medaka>). Homopolymer indels in sequences were manually edited using the RABV (SADB19 M31046) as reference in Bioedit7.2.5. Multiple sequence alignments were accomplished by Clustal Omega 1.2.2 on the Geneious Prime 10.2.2 program (Geneious Prime 2020.2.4 ([https://www.geneious.com](http://www.geneious.com/))).

Due to variability in the available RABV sequence data from Haiti and Dominican Republic, several phylogenetic analyses were performed. To unearth insights into the relationship between rabies isolates collected in Haiti and the Dominican Republic, a dataset of 133 complete and partial N gene sequences from Hispaniola and 3 outgroup sequences from Unites States raccoon and South Central skunk rabies variants were analyzed on Bayesian Evolutionary Analysis Sampling Trees BEAST 2.5 (7) using parameter-rich model GTR+I+G model (8) with the strict and relaxed molecular clock and coalescent constant population. Convergence across runs and 100 million generations sampling every 10,000 generations; burn-in times were corroborated using Tracer (9). Final maximum clade credibility tree (MCC) was generated by TreeAnnotator and visualized on Figtree v1.4.4 (10). To understand the spatial distribution and movements of rabies virus across the Hispaniola Island, the metadata information of each sequence which contained the commune or district level geographic location along with the sub-lineages identified in the phylogenetic tree using the strict clock model were colored and plotted onto the geographic map using QGIS version 3.40.4 with administrative boundaries from geoBoundaries and basemap from US Geological Survey (10-12).

To determine the relationship of the Hispaniola rabies virus sequences to others, a reduced set of 90 complete nucleoprotein gene sequences from Hispaniola with 81 representative sequences (Supplemental table 2) of major Cosmopolitan dog rabies clades were included. The temporal signal were examined using Tempest v1.5.3 (11) and the phylogenetic analysis was performed using GTR+I+G model with a strict and relaxed clock with and without sampling dates on BEAST2, Temporal signal comparison of both analysis can be found at supplemental table 3. Due to the 95% HPD higher posterior values, the phylogenetic produced by the strict clock analysis was used to draw the figure 2. . A sub tree (Figure 3) that included CAR1, AM1 and CAR2 was draw to visualize the TMRCA of the RABVs included in this study. Additionally, we manually transcribed and aligned the partial nucleoprotein sequences (200bp) for four samples (Human -87DRHM133, Dog -87DRDG1334, and two mongoose - 87DRMG628, 87DRMG629) that were previously documented (12). The length of the sequence corresponded to positions 1087 to 1286 on the N gene.

# Supplementary Figures and Tables

## Supplementary Figure 1. Maximum clade credibility tree using the 200 bp N gene. The colored taxon labels indicate the lineage/sub-lineage of the CAR1 subclade. Within the sub-lineage CAR1a-C and lineage CAR1b sequences transcribed manually from a previous study are labeled in black. The number in the branches is the posterior support, and the number in the bar is the substitution per site.

**
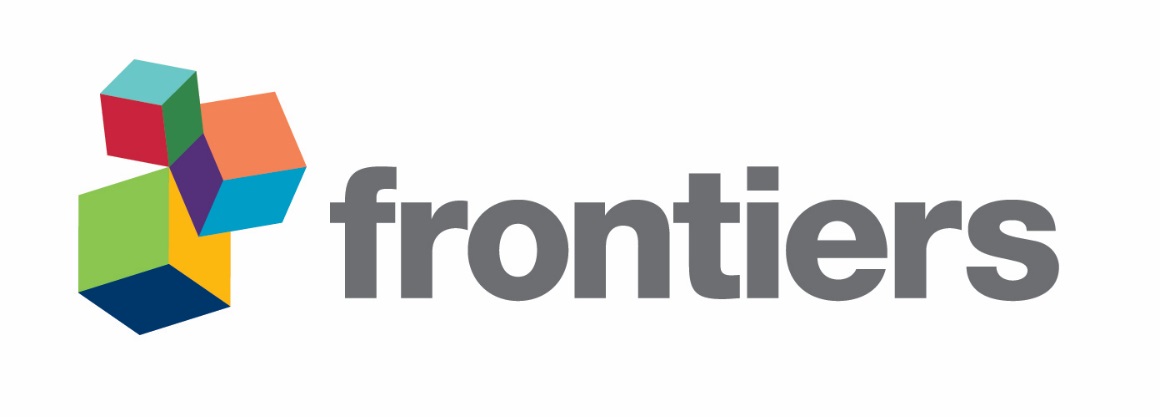
**

**Disclaimer**. Use of trade names and commercial sources in this manuscript is for identification only and does not imply endorsement by the Centers for Disease Control and Prevention (CDC), the Public Health Service, or the U.S. Department of Health and Human Services. Product names, brands, and other trademarks referred are the property of their respective trademark holders. These trademark holders are not affiliated with CDC. CDC does not sponsor or endorse these materials but references them as examples of materials used in this research.

**Reference**

1. Gigante CM, Dettinger L, Powell JW, Seiders M, Condori REC, Griesser R, et al. Multi-site evaluation of the LN34 pan-lyssavirus real-time RT-PCR assay for post-mortem rabies diagnostics. PloS one. 2018;13(5):e0197074.

2. Wadhwa A, Wilkins K, Gao J, Condori Condori RE, Gigante CM, Zhao H, et al. A Pan-Lyssavirus Taqman Real-Time RT-PCR Assay for the Detection of Highly Variable Rabies virus and Other Lyssaviruses. PLoS neglected tropical diseases. 2017 Jan;11(1):e0005258.

3. Markotter W, Kuzmin I, Rupprecht CE, Randles J, Sabeta CT, Wandeler AI, et al. Isolation of Lagos bat virus from water mongoose. Emerging infectious diseases. 2006 Dec;12(12):1913-8.

4. Condori-Condori RE, Streicker DG, Cabezas-Sanchez C, Velasco-Villa A. Enzootic and epizootic rabies associated with vampire bats, peru. Emerging infectious diseases. 2013;19(9).

5. Gigante CM, Yale G, Condori RE, Costa NC, Long NV, Minh PQ, et al. Portable Rabies Virus Sequencing in Canine Rabies Endemic Countries Using the Oxford Nanopore MinION. Viruses. 2020 Nov 4;12(11).

6. Hall TA. BioEdit: a user-friendly biological sequence alignment editor and analysis program for Windows 95/98/NT. Nucleic Acids Symp; 1999. p. 95 8.

7. Bouckaert R, Vaughan TG, Barido-Sottani J, Duchêne S, Fourment M, Gavryushkina A, et al. BEAST 2.5: An advanced software platform for Bayesian evolutionary analysis. PLoS computational biology. 2019;15(4):e1006650.

8. Abadi S, Azouri D, Pupko T, Mayrose I. Model selection may not be a mandatory step for phylogeny reconstruction. Nature communications. 2019 2019/02/25;10(1):934.

9. Rambaut A, Drummond AJ, Xie D, Baele G, Suchard MA. Posterior Summarization in Bayesian Phylogenetics Using Tracer 1.7. Systematic Biology. 2018;67(5):901-4.

10. Rambaut A. A FigTree version 1.4.0. Computer sofware distributed by the autor. <http://tree.bio.ed.ac.uk/software/figtree/>; 2009.

11. Rambaut A, Lam TT, Max Carvalho L, Pybus OG. Exploring the temporal structure of heterochronous sequences using TempEst (formerly Path-O-Gen). Virus Evolution. 2016;2(1).

12. Smith JS, Orciari LA, Yager PA, Seidel HD, Warner CK. Epidemiologic and historical relationships among 87 rabies virus isolates as determined by limited sequence analysis. The Journal of infectious diseases. 1992 Aug;166(2):296-307.
