# Supplementary figures and images for: Using molecular approaches to assess rabies virus diversity in Haiti and the Dominican Republic
Source: Front Microbiol. 2026 Jan 20;16:1688184. doi: 10.3389/fmicb.2025.1688184 (PMC12864505; doi:10.3389/fmicb.2025.1688184)

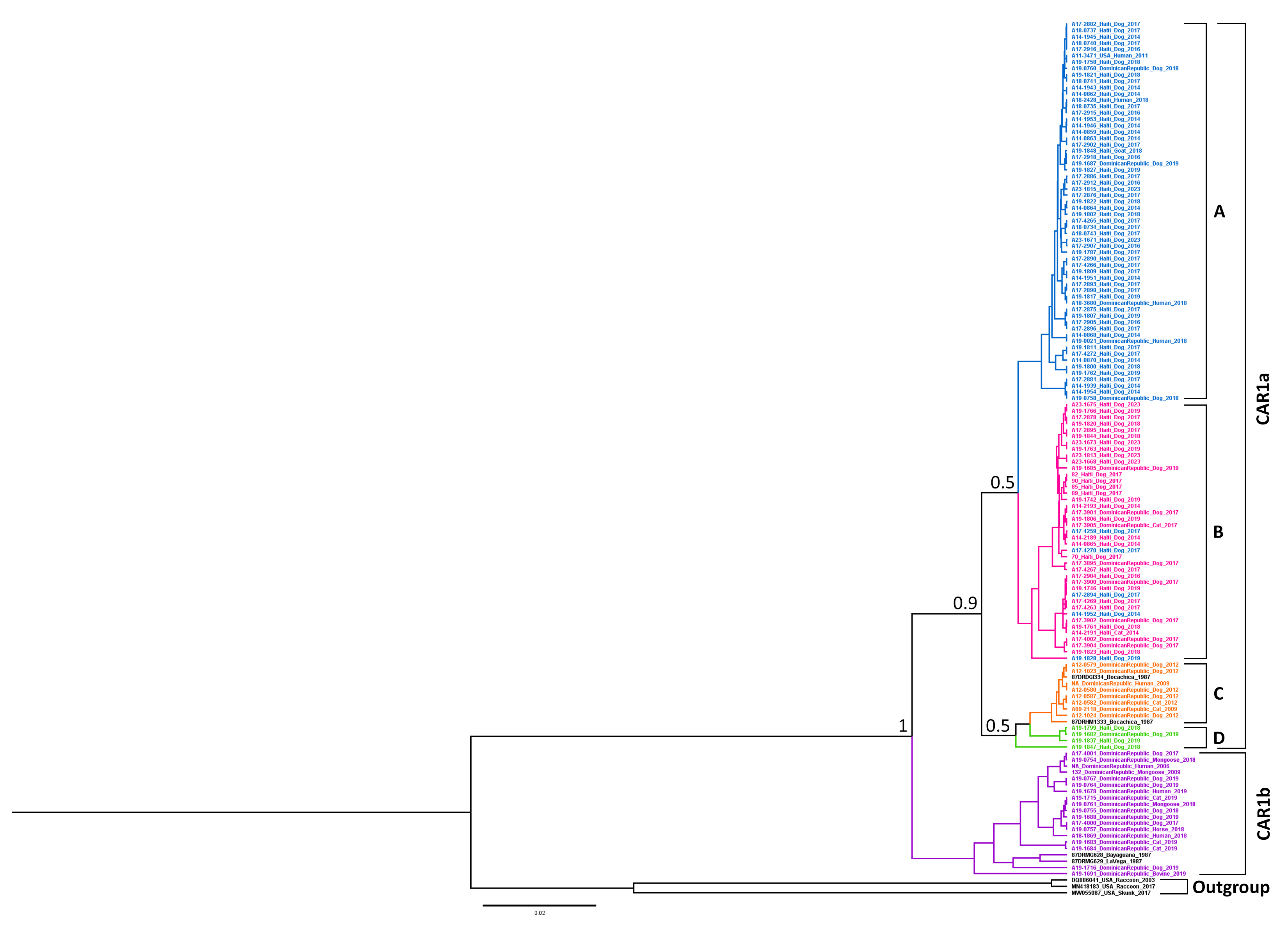

Supplement: Supplementary file 2 [file Image_1.jpeg]
